# Inhibitory Effect of a Glutamine Antagonist on Proliferation and Migration of VSMCs via Simultaneous Attenuation of Glycolysis and Oxidative Phosphorylation

**DOI:** 10.3390/ijms22115602

**Published:** 2021-05-25

**Authors:** Hyeon Young Park, Mi-Jin Kim, Seunghyeong Lee, Jonghwa Jin, Sungwoo Lee, Jung-Guk Kim, Yeon-Kyung Choi, Keun-Gyu Park

**Affiliations:** 1 Department of Biomedical Science, Graduate School, Kyungpook National University, Daegu 41566, Korea; coolphyp@gmail.com (H.Y.P.); ngell92@gmail.com (S.L.); 2 BK21 FOUR KNU Convergence Educational Program of Biomedical Sciences for Creative Future Talents, School of Medicine, Kyungpook National University, Daegu 41566, Korea; 3 Department of Internal Medicine, School of Medicine, Kyungpook National University, Kyungpook National University Hospital, Daegu 41944, Korea; kij200@nate.com (M.-J.K.); becauseofu77@gmail.com (J.J.); jugkim@knu.ac.kr (J.-G.K.); 4 Research Institute of Aging and Metabolism, Kyungpook National University, Daegu 41566, Korea; 5 New Drug Development Center, Daegu Gyeongbuk Medical Innovation Foundation, Daegu 41061, Korea; swlee@dgmif.re.kr

**Keywords:** glutamine antagonist, vascular smooth muscle cells, glycolysis, oxidative phosphorylation, mTORC1

## Abstract

Excessive proliferation and migration of vascular smooth muscle cells (VSMCs) contribute to the development of atherosclerosis and restenosis. Glycolysis and glutaminolysis are increased in rapidly proliferating VSMCs to support their increased energy requirements and biomass production. Thus, it is essential to develop new pharmacological tools that regulate metabolic reprogramming in VSMCs for treatment of atherosclerosis. The effects of 6-diazo-5-oxo-L-norleucine (DON), a glutamine antagonist, have been broadly investigated in highly proliferative cells; however, it is unclear whether DON inhibits proliferation of VSMCs and neointima formation. Here, we investigated the effects of DON on neointima formation in vivo as well as proliferation and migration of VSMCs in vitro. DON simultaneously inhibited FBS- or PDGF-stimulated glycolysis and glutaminolysis as well as mammalian target of rapamycin complex I activity in growth factor-stimulated VSMCs, and thereby suppressed their proliferation and migration. Furthermore, a DON-derived prodrug, named JHU-083, significantly attenuated carotid artery ligation-induced neointima formation in mice. Our results suggest that treatment with a glutamine antagonist is a promising approach to prevent progression of atherosclerosis and restenosis.

## 1. Introduction

Vascular smooth muscle cells (VSMCs) are the major component of the vasculature and help to maintain vessel tone, the bloodstream, and blood pressure [1]. Fully differentiated VSMCs remain in a non-proliferative state under normal conditions, while VSMCs dedifferentiate and their proliferation rate increases upon vascular injury [2,3]. Phenotypic switching of VSMCs in response to various physiological and pathological factors has long been considered of fundamental importance for proliferation and migration of VSMCs and pathological intima formation, which leads to the development of various vascular diseases including atherosclerosis, transplant vasculopathy, and pulmonary hypertension [4,5]. Thus, better understanding of the underlying mechanism might lead to the identification of a new therapeutic target for neointimal hyperplasia.

Under physiological conditions, VSMCs undergo metabolic reprogramming such as enhancement of aerobic glycolysis to support their increased energy requirements and biosynthesis of macromolecules during rapid proliferation and contractile switching [6,7,8]. Previous studies demonstrated that enhanced glycolysis is highly required during proliferation of VSMCs by showing that glycolytic enzyme expression, lactate production, and glucose utilization are increased in growth factor-stimulated VSMCs [9,10,11,12]. Rapidly proliferating VSMCs also have an increased demand for glutamine, which is the most abundant circulating nutrient and is needed to maintain the redox balance, amino acid production, nucleotide synthesis, and extracellular matrix production [13,14]. Increasing evidence shows that enhanced glycolysis and glutaminolysis contribute to proliferation, migration, and collagen synthesis of vascular cells [15,16]. Therefore, therapeutic strategies based on the blockade of aerobic glycolysis or glutamine metabolism may be effective for treatment of occlusive vascular diseases.

6-diazo-5-oxo-L-norleucine (DON) is an L-glutamine antagonist that was discovered in the 1950s as a natural product of Streptomyces bacteria [17]. DON robustly inhibits cell proliferation by blocking glutamine-dependent enzyme pathways including those involving glutaminase 1 [18]. Despite the efficacy of DON in preclinical and clinical studies, its substantial peripheral toxicity has hampered its approval as a chemotherapeutic agent [18,19]. On the other hand, ethyl 2-(2-amino-4-methylpentanamido)-DON (JHU-083) is a DON-derived prodrug [20,21]. This inert glutamine antagonist is activated via proteolytic cleavage by enriched enzymes such as cathepsin-L in vivo, leading to the release of active DON [20,22]. Thus, JUH-083 exhibits increased oral bioavailability and decreased toxicity [21]. Although the anti-proliferative effects of glutamine antagonists have been broadly investigated in many types of cancer [20,23,24], their effects on proliferation of VSMCs and ability to halt restenosis remain to be elucidated.

In this study, we investigated whether the glutamine antagonist DON and the DON-derived prodrug JHU-083 inhibit proliferation and migration of VSMCs and suppress neointima formation in a mouse carotid artery ligation model.

## 2. Results

### 2.1. DON Attenuates Fetal Bovine Serum (FBS)- and Platelet-Derived Growth Factor (PDGF)-Induced Upregulation of Mitochondrial Respiration in VSMCs

**Figure 1:**
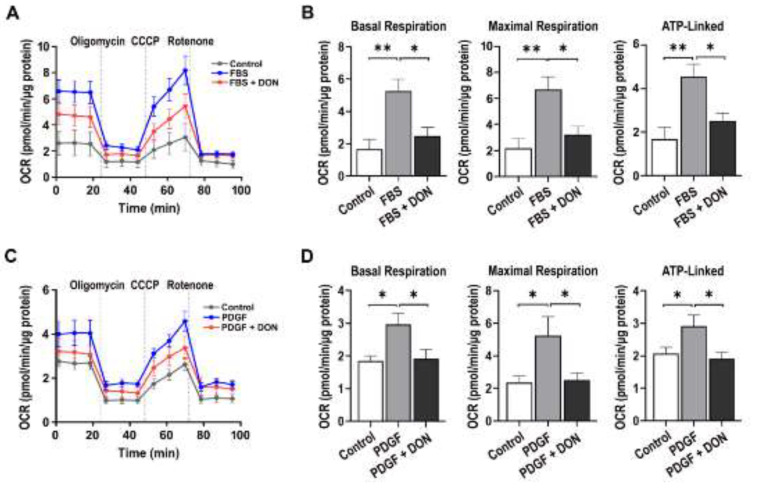
DON reduces mitochondrial respiration in growth factor-stimulated VSMCs. (**A**) and (**C**) OCR kinetic traces in FBS (**A**)- and PDGF (**C**)-stimulated VSMCs treated with or without DON. (**B**) and (**D**) Rates of basal, maximal, and ATP-linked respiration in FBS (**B**)- and PDGF(**D**)-stimulated VSMCs treated with or without DON. Data are expressed as the mean ± SEM (*n* = 3 technical replicates). * *p* < 0.05 and ** *p* < 0.01.

Given that glutamine contributes to TCA intermediates and DON targets mitochondria in cancer research [25], we first measured the oxygen consumption ratio (OCR) in DON-treated VSMCs stimulated with FBS or PDGF. Basal, maximal, and ATP-linked respiration was increased upon FBS or PDGF stimulation; however, DON significantly decreased all these respiratory parameters (Figure 1A–D).

### 2.2. DON Attenuates FBS- and PDGF-Induced Upregulation of Mammalian Target of Rapamycin Complex I (mTORC1) Activity and Glycolysis in VSMCs

**Figure 2:**
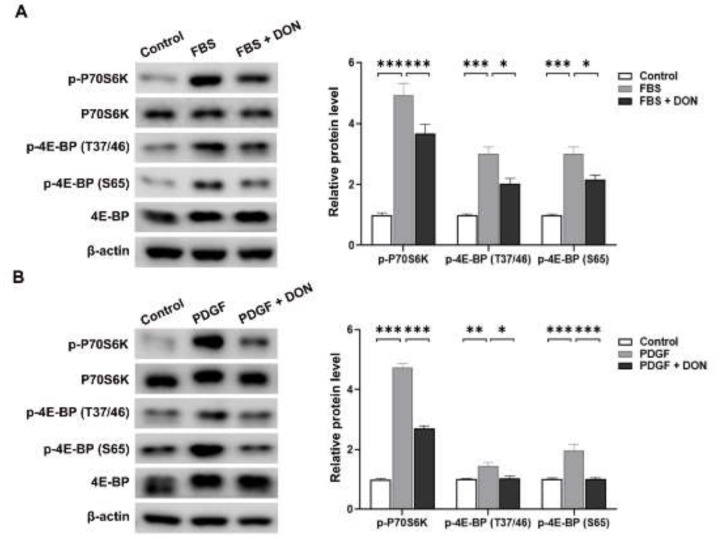
DON attenuates mTORC1 activity in growth factor-stimulated VSMCs. (**A**) and (**B**) Representative western blots showing the effects of DON on the levels of p-p70S6K, p-70S6K, 4E-BP, p-4E-BP (Thr37/46), and p-4E-BP (Ser65) in VSMCs stimulated with FBS (**A**) and PDGF (**B**). Data are expressed as the mean ± SEM (*n* = 3 technical replicates). * *p* < 0.05, ** *p* < 0.01, and *** *p* < 0.001.

**Figure 3:**
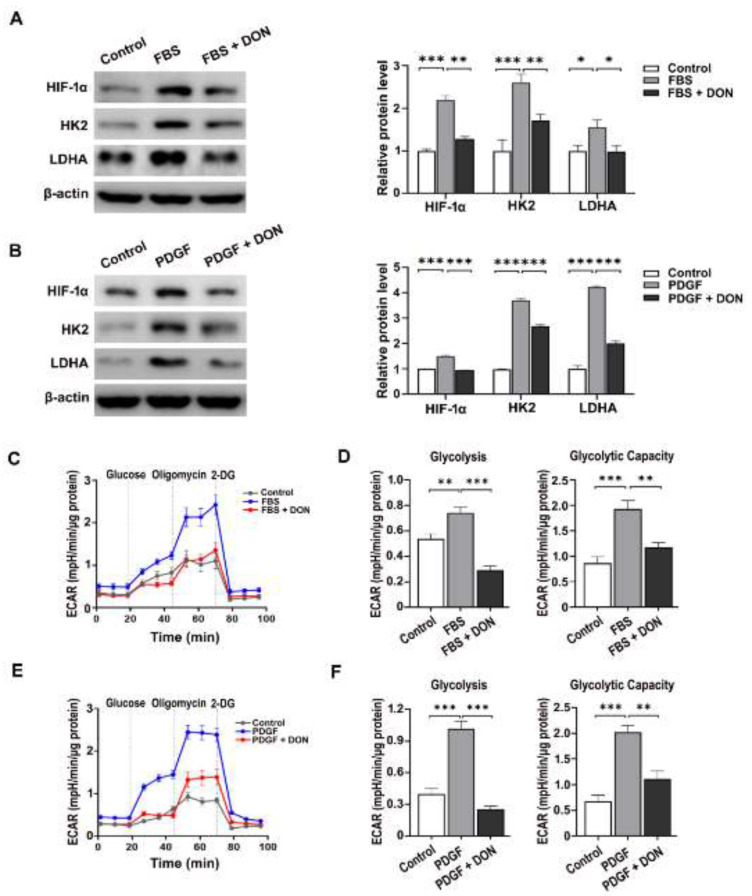
DON suppresses glycolysis in growth factor-stimulated VSMCs. (**A**) and (**B**) Representative western blots showing the effects of DON on the levels of HIF-1α, HK2, and LDHA in VSMCs stimulated with FBS (**A**) and PDGF (**B**). Data in bar graphs are mean ± SEM of three independent measurements. (**C**)–(**F**) The ECAR in FBS- and PDGF-stimulated VSMCs. ECAR kinetic traces and the glycolytic rate and glycolytic capacity of FBS (**C**) and (**D**)- and PDGF (**E**) and (**F**)- stimulated VSMCs treated with or without DON. Data are expressed as the mean ± SEM (*n* = 3 technical replicates). * *p* < 0.05, ** *p* < 0.01, and *** *p* < 0.001.

Glutaminolysis regulates activation of mTORC1 [26]. Thus, we investigated whether DON regulates mTORC1 activity in cultured VSMCs by measuring phosphorylation of 70 kDa ribosomal protein S6 kinase (p70S6K) and eukaryotic initiation factor 4E-binding protein 1 (4E-BP1), which are downstream substrates of mTORC1. DON attenuated the FBS- and PDGF-induced increases in p-p70S6K and p-4E-BP1 expression (Figure 2A,B). Given that mTORC1 increases glucose uptake and glycolysis by upregulating hypoxia-inducible factor-1α (HIF-1α) [27], we next investigated whether the DON-induced suppression of mTORC1 inhibits glycolysis in VSMCs. The levels of HIF-1α and its downstream targets hexokinase 2 (HK2) and lactate dehydrogenase A (LDHA) were increased in FBS- and PDGF-stimulated VSMCs, and downregulated by DON (Figure 3A,B). Consistent with the downregulation of HIF-1α, HK2, and LDHA, DON suppressed glycolysis and the glycolytic capacity in FBS- and PDGF-simulated VSMCs, as determined by measuring the extracellular acidification rate (ECAR) (Figure 3C–F). Overall, these results indicate that blockade of glutamine using DON significantly inhibits mTORC1 activity and HIF-1α expression, which in turn suppresses glycolysis in growth factor-stimulated VSMCs.

### 2.3. DON Delays Cell Cycle Progression and Inhibits Proliferation and Migration of FBS- and PDGF-Stimulated VSMCs

**Figure 4:**
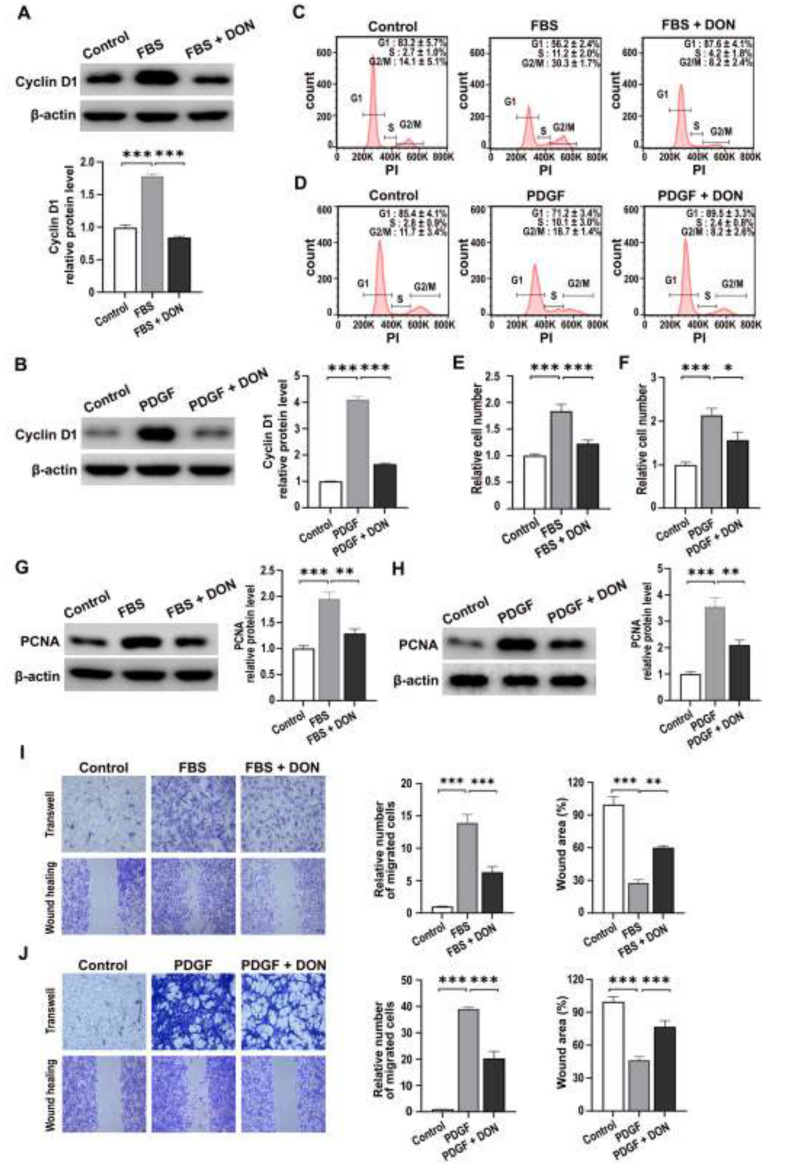
DON delays growth factor-induced cell cycle progression and inhibits proliferation and migration of VSMCs. (**A**,**B**) Representative western blots showing the effects of DON on the level of cyclin D1 in VSMCs stimulated with FBS (**A**) and PDGF (**B**). Data in bar graphs are mean ± SEM of three independent measurements. (**C**,**D**) Histogram showing the cell cycle distribution of VSMCs stimulated with FBS (**C**) and PDGF (**D**). (**E**,**F**) Effects of DON on proliferation of VSMCs stimulated with FBS (**E**) and PDGF (**F**). (**G**,**H**) Representative western blots showing the effects of DON on the level of PCNA in VSMCs stimulated with FBS (**G**) and PDGF (**H**). Data in bar graphs are mean ± SEM of three independent measurements. (**I**,**J**) Transwell migration (upper) and wound healing assays (lower) showing the effects of DON on migration of VSMCs stimulated with FBS (**I**) and PDGF (**J**). Data in bar graphs are mean ± SEM of three independent measurements. * *p* < 0.05, ** *p* < 0.01, and *** *p* < 0.001.

mTORC1 regulates cell cycle progression [28,29]; therefore, we investigated whether DON affects cell cycle progression of FBS- and PDGF-stimulated VSMCs. Western blot analysis revealed that the level of cyclin D1, a key regulator of VSMC proliferation, increased in response to FBS and PDGF, and this effect was reversed by DON (Figure 4A,B). Furthermore, flow cytometric analysis of the cell cycle showed that DON attenuated FBS- and PDGF-stimulated progression from G1 to S phase (Figure 4C,D).

Given that DON inhibited FBS- and PDGF-stimulated mitochondrial respiration, mTORC1 activity, and glycolysis, we next investigated its effects on proliferation and migration of VSMCs. As expected, DON significantly reduced FBS- and PDGF-stimulated proliferation of VSMCs and the level of proliferating cell nuclear antigen (PCNA) (Figure 4E–H). Wound healing and Transwell chamber assays showed that DON markedly attenuated FBS- and PDGF-stimulated migration of VSMCs (Figure 4I,J).

### 2.4. JHU-083 Attenuates Carotid Artery Ligation-Induced Neointimal Hyperplasia

**Figure 5:**
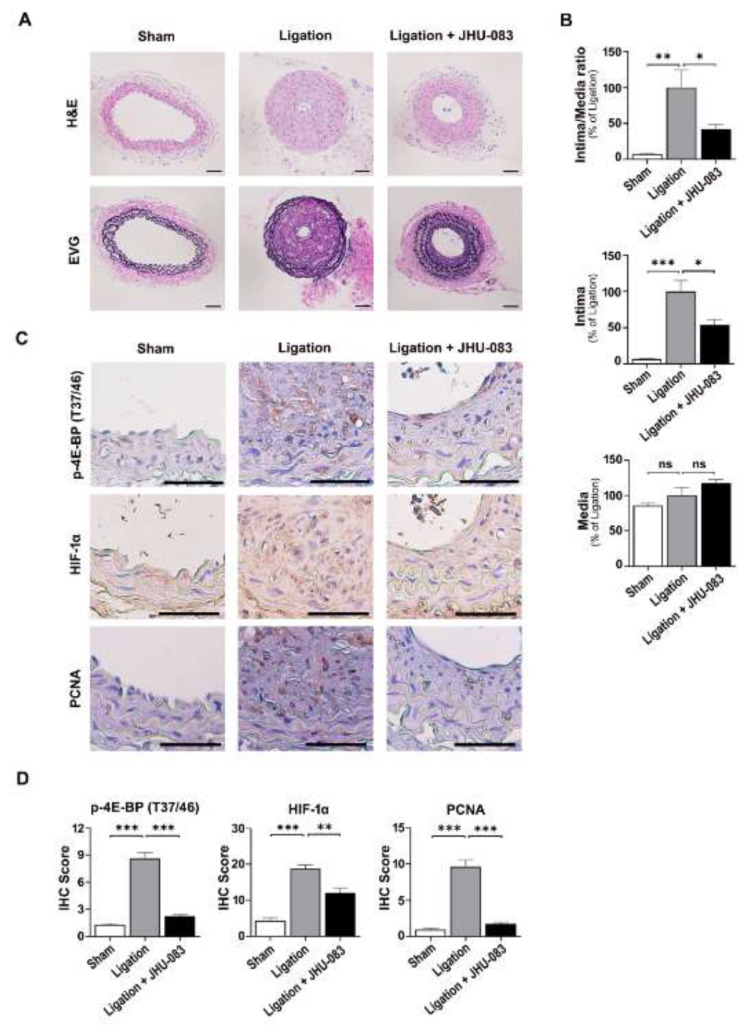
JHU-083 attenuates carotid artery ligation-induced neointimal hyperplasia. (**A**) Representative cross-sections of mouse carotid arteries stained with H&E and Elastic-van Gieson (EVG), Scale bar represents 50 µm. (**B**) The relative ratios of the neointimal and medial areas, the intimal area, and the medial area quantified by morphometric analysis. Data are expressed as the mean  ±  SEM (*n* = 4–5 per group). (**C**) Immunohistochemical staining of PCNA, p-4E-BP(Thr37/46) and HIF-1α in mouse carotid arteries. (**D**) Cells immunohistochemically positive for PCNA, p-4E-BP(Thr37/46), and HIF-1α in arteries were quantified. Data are expressed as mean  ±  SEM (*n* = 4–5 per group). Scale bar represents 50 µm. NS, not significant; * *p* < 0.05, ** *p* < 0.01, and *** *p* < 0.001.

Finally, we investigated whether JHU-083, a DON-derived prodrug, affects proliferation and migration of VSMCs after carotid artery ligation in mice. Representative cross-sections of arteries showed neointima formation and substantial narrowing of the arterial lumen after carotid artery ligation for 4 weeks. The intimal area was significantly smaller and the ratio of the neointimal layer to the medial layer was significantly lower in the JHU-083-treated group than in the ligation only group (Figure 5A,B). Consistent with the effects of DON in vitro, JHU-083 decreased p-4E-BP(T37/46), HIF-1α, and PCNA staining in arteries after carotid artery ligation (Figure 5C,D).

## 3. Discussion

Our study revealed that the glutamine antagonist DON markedly inhibits growth factor-stimulated proliferation and migration of VSMCs. We demonstrated that the efficacy of DON was attributable to simultaneous suppression of FBS- or PDGF-stimulated glycolysis and oxidative phosphorylation as well as downregulation of mTORC1 activity in VSMCs. Finally, we showed that administration of JHU-083 attenuated neointimal hyperplasia after carotid artery ligation in mice.

mTORC1 is a master cell growth factor, and an inhibitor of this protein and rapamycin are commonly used in clinical settings to control VSMCs overgrowth-induced restenosis [28,30]. Glutaminolysis has a dual role in cancer cells; it sustains the TCA cycle and activates mTORC1 [26]. Inhibition of glutaminolysis by DON prevents GTP loading of RagB and lysosomal translocation and subsequent activation of mTORC1 [26]. In contrast to the inhibitory effect of DON on glutaminolysis, a more recent study reported that JHU-083 reduces glioma cell proliferation by disrupting mTORC1 signaling, but the effect is independent of glutaminolysis [31]. Consistent with these previous results showing the inhibitory effects of a glutamine antagonist on mTORC1 activity, we observed that DON abrogated growth factor-induced mTORC1 activity in VSMCs. mTORC1-induced signaling pathways also regulate cyclin D1 protein expression and cell cycle progression from G to S phase independent of gene transcription [29,32]. Based on our finding that DON decreased cyclin D1 expression and induced cell cycle arrest, the reduction of growth factor-stimulated VSMC growth is attributable to disruption of mTORC1 by DON. Although the glutamine antagonist JHU-083 prevented ligation-induced occlusion, it did not completely restore the overall organization of the tissue induced by ligation. Considering that a previous report showed that DON and rapamycin had an additive effect on lymphocyte proliferation, with subsequent suppression of arthritis in SKG mice [33] it is expected that JHU-083 and rapamycin may additively affect the inhibition of VSMC proliferation and migration in vitro and in vivo. Furthermore, given that the mTOR pathway is implicated in stress fiber formation and focal adhesion formation [34,35], it might be worthwhile to investigate whether DON affects FBS- and PDGF-induced changes in stress fibers and focal adhesion formation in VSMCs.

Increased glycolysis appears to be critical for the bioenergetic shift that occurs during proliferation and migration of VSMCs [8,10]. Growth factor-treated VSMCs exhibit enhanced glycolytic flux and express markers of the synthetic VSMC phenotype [5,36,37]. We previously identified LDHA, which catalyzes the final step of glycolysis, as a therapeutic target for uncontrolled proliferation and migration of VSMCs [38]. Considering that mTORC1 reinforces the glycolytic program by increasing translation of HIF-1α mRNA and stabilizing HIF-1α expression [39], we assume that inhibition of mTORC1 by DON suppresses glycolysis in VSMCs in response to FBS or PDGF. We showed that upregulation of HIF-1α and glycolysis in response to growth factor stimulation was attenuated by glutamine blockade. Together, our results demonstrate that glutamine blockade inhibits not only mTORC1 activity but also HIF-1α expression, which in turn downregulates transcription of HK2 and LDHA [7,36]. Considering that proliferation of VSMCs and neointima formation are dependent on activation of glutamine uptake [40], simultaneous reduction of glutaminolysis and glycolysis may be a promising strategy to ameliorate constriction of vessel lumens during atherosclerosis and restenosis.

In conclusion, the present study shows that the glutamine antagonist DON inhibits proliferation and migration of growth factor-stimulated VSMCs by simultaneously suppressing FBS- and PDGF-stimulated glycolysis and oxidative phosphorylation. This study suggests that glutamine antagonists can be used to treat vascular occlusive disease.

## 4. Materials and Methods

### 4.1. Murine Model of Carotid Artery Ligation

Carotid artery ligation-induced neointimal hyperplasia was established in male C57BL/6J mice, as previously described [41]. Briefly, the unilateral common carotid artery was surgically ligated with a 5.0 suture tied near the distal bifurcation. JHU-083 was dissolved in sterile phosphate-buffered saline (PBS) immediately before treatment for in vivo studies [42]. Mice were administered JHU-083 via oral gavage (0.6 mg/kg/day, 6 days per week for 4 weeks). Arteries proximal to the ligation site were analyzed by hematoxylin and eosin (H&E) staining and an Elastic-van Gieson (EVG) staining (Abcam, Cambridge, UK). The cross-sectional intimal and medial areas were quantified using ImageJ software (National Institutes of Health, Bethesda, MD, USA). The intima-to-media ratio was calculated from the mean of these measurements.

### 4.2. Immunohistochemistry

Immunohistochemistry (IHC) was performed on formalin-fixed, paraffin-embedded tissue sections, as previously described [38]. The sections were incubated with anti-PCNA, (1:1000, Cell Signaling Technology, Beverly, MA, USA) anti-p-4E-BP1 (Thr37/46) (1:1000, Cell Signaling Technology) and anti-HIF-1α (1:500, R&D Systems, Minneapolis, MN, USA). The sections were visualized with diaminobenzidine (Liquid DAB+ Substrate Chromogen System; Dako, Carpinteria, CA, USA) and counterstained with Mayer’s hematoxylin (Merck Inc., Darmstadt, Germany) at room temperature for 40 s. Images of each group were obtained using a light microscope with CellSens Entry 1.9 imaging system (Olympus Corporation, Tokyo, Japan). Immunopositive cells and/or areas are shown in brown.

### 4.3. Cell Culture

VSMCs were isolated from the aortas of male Sprague–Dawley rats (weight, 90–100 g) according to a previously published method [43]. Briefly, the aortic tissues were carefully harvested and cut into pieces. The tissue pieces were immersed in low-glucose Dulbecco’s modified Eagle’s medium (DMEM; Hyclone, South Logan, UT, USA) containing 20% FBS (Hyclone) and 1% penicillin/streptomycin at 37 °C with 5% CO_2_. Non-adherent cells were removed by replacing the medium every other day. All VSMCs used in this study were between the fourth and ninth passages.

### 4.4. Cell Counting

Primary VSMCs were cultured for 18 h under serum-starved conditions and then incubated for 24 h in the presence or absence of 10% FBS or PDGF-BB (20 ng/mL) with 5 μM DON (Selleckchem, Houston, TX, USA). Cells were harvested and the total cell numbers were determined by the trypan blue exclusion assay using a hemocytometer under a microscope.

### 4.5. Western Blot Analysis

Western blot analysis was performed as previously described [44]. Proteins in cell lysates were separated by SDS-PAGE and transferred to PVDF membranes (Millipore, MA, USA). The membranes were incubated with primary antibodies specific for the following proteins: p-p70S6K (Thr389), p70S6K, p-4E-BP1 (Ser65), p-4E-BP1 (Thr37/46), 4E-BP1, PCNA, cyclin D1, LDHA, and HK2 (Cell Signaling Technology, Beverly, MA, USA); HIF-1α (R&D Systems, Minneapolis, MN, USA); and β-actin (Sigma, Saint Louis, MO, USA). After three washes in TBST, membranes were incubated with horseradish peroxidase-conjugated secondary antibodies (Santa Cruz Biotechnology, Dallas, TX, USA). Immunoreactive bands were detected with enhanced chemiluminescence reagent (BioNote, Gyeonggi-do, Korea) and visualized using an Amersham Imager 600 (GE Healthcare, Chicago, IL, USA). Band intensity was quantified using Image J software (National Institutes of Health, Bethesda, MD, USA).

### 4.6. Migration Assays

For the wound healing assay, VSMCs (1 × 10^5^ cells) were plated onto 6-well plates and serum-starved for 18 h. An artificial wound (scratch) was generated using a 200 μL pipette tip. The cells were incubated with or without 5 μM DON for 24 h in the presence or absence of 10% FBS or PDGF-BB (20 ng/mL). When the wound had closed, cells were fixed in 4% paraformaldehyde and stained with 0.05% crystal violet. For the Transwell migration assay, VSMCs (1 × 10^4^ cells) were seeded onto the microporous membrane (8.0 µm) in the upper chamber of the Transwell^®^ (Corning Incorporated, New York, NY, USA). Cells were serum-starved for 18 h and then incubated with or without 5 μM DON for 24 h in the presence or absence of 20% FBS or PDGF-BB (20 ng/mL). Cells that had not migrated in the upper chamber were gently removed using a cotton swab. Cells that had migrated through the membrane to the lower chamber were fixed in methanol and stained with 0.05% crystal violet. Quantification of the transwell migration and wound area were performed using Image J software (National Institutes of Health, Bethesda, MD, USA).

### 4.7. Flow Cytometric Analysis

Cells were synchronized in G1 phase by serum starvation for 6 h, and then incubated with or without 5 μM DON for 12 h in the presence or absence of 10% FBS or PDGF-BB (20 ng/mL). Thereafter, cells were trypsinized, washed with cold PBS containing 2% FBS, fixed for 1 h in 70% cold (−20 °C) ethanol, and stained for 30 min with PI/RNase Staining Buffer (BD Pharmingen, NJ, USA) in the dark. The percentage of cells in each phase of the cell cycle (G1, S, and G2/M) was analyzed using an Accuri™ C6 Plus flow cytometer (BD Bioscience, San Jose, CA, USA). Data were analyzed using FlowJo V10 software (Tree Star, Inc., Ashland, OR, USA).

### 4.8. Measurement of the OCR and ECAR

The bioenergetic properties of primary VSMCs under different conditions were determined using a XF-24 Seahorse extracellular flux analyzer (Seahorse Bioscience, North Billerica, MA, USA). Cells were seeded into a Seahorse XF24 plate, starved for 18 h, and then incubated for 24 h with or without 5 μM DON in the presence or absence of 10% FBS or PDGF-BB (20 ng/mL). For the Mito Stress assay, the culture medium was replaced by Seahorse XF DMEM supplemented with 5.55 mM glucose and 1 mM sodium pyruvate, and cultures were pre-incubated for 1 h at 37 °C in a CO_2_-free incubator before measurements. Changes in cellular respiration were assessed over time upon consecutive injections of 1 µM oligomycin (Sigma, Saint Louis, MO, USA), 2 µM carbonyl cyanide 3-chlorophenylhydrazone (CCCP; Sigma, Saint Louis, MO, USA), and 1 µM rotenone (Sigma, Saint Louis, MO, USA) at the indicated timepoints. For the Glycolysis Stress assay, the culture medium was replaced by Seahorse XF DMEM containing 5.55 mM glucose before measurements. Cultures were equilibrated for 1 h at 37 °C in a CO_2_-free incubator immediately before the XF assay. pH changes of the medium were determined in real-time upon sequential treatment with 10 mM glucose (Sigma), 1 μM oligomycin (Sigma, Saint Louis, MO, USA), and 100 mM 2-deoxyglucose (2-DG; Sigma, Saint Louis, MO, USA). The OCR and ECAR were calculated automatically using the program of the Seahorse XF Mito Stress Test and Seahorse XF Glycolysis Stress Test according to the manufacturer’s protocol, respectively.

### 4.9. Statistical Analysis

All values in the graphs represent the mean ± standard error of the mean (SEM). A one-way analysis of variance followed by Dunnett’s multiple comparison test was used to assess differences between groups (GraphPad Prism 8.0, San Diego, CA, USA).

## Data Availability

Not applicable.
